# Crossing the lipid divide

**DOI:** 10.1016/j.jbc.2021.100859

**Published:** 2021-06-05

**Authors:** Christian Sohlenkamp

**Affiliations:** Centro de Ciencias Genómicas, Universidad Nacional Autónoma de México, Cuernavaca, Morelos, Mexico

**Keywords:** cardiolipin, bacterial membranes, synthetic biology, archaeal lipids, cardiolipin synthase, AG, archaetidylglycerol, CDP-DAG, cytidine diphosphate-diacylglycerol, CL, cardiolipin, G1P, glycerol-1-phosphate, G3P, glycerol-3-phosphate, PG, phosphatidylglycerol, PLD, phospholipase D

## Abstract

Archaeal membrane lipids are structurally different from bacterial and eukaryotic membrane lipids, but little is known about the enzymes involved in their synthesis. In a recent study, Exterkate *et al.* identified and characterized a cardiolipin synthase from the archaeon *Methanospirillum hungatei*. This enzyme can synthesize archaeal, bacterial, and mixed archaeal/bacterial cardiolipin species from a wide variety of substrates, some of which are not even naturally occurring. This discovery could revolutionize synthetic lipid biology, being used to construct a variety of lipids with nonnatural head groups and mixed archaeal/bacterial hydrophobic chains.

Lipid bilayer membranes are formed of amphiphilic lipids and embedded proteins. In most cases and conditions studied, these amphiphilic lipids are glycerophospholipids, composed of two hydrophobic tails, a glycerol moiety, a phosphate group, and a variable head group (1). Bacteria/Eukarya produce membrane lipids with hydrophobic fatty acid tails that are ester-bound to glycerol-3-phosphate (G3P), whereas Archaea synthesize membrane lipids with hydrophobic isoprenoid lipid tails that are ether-linked to glycerol-1-phosphate (G1P) with the opposite chirality. This stereochemical difference between Bacteria/Eukarya and Archaea is called the “lipid divide,” and at present, we do not have a good understanding of why this difference evolved (2). The structures of many bacterial and eukaryotic membrane lipids and the enzymes involved in their synthesis have been investigated mainly using *Escherichia coli* and *Saccharomyces cerevisiae* (3, 4). Only in recent years have these aspects been studied in a wider variety of Bacteria/Eukarya model organisms. On the other hand, very little is known about the enzymes involved in membrane lipid synthesis in Archaea.

Cardiolipin (CL) is an anionic membrane lipid almost ubiquitous in bacteria and throughout the eukaryotic kingdoms. Distinct from other phospholipids, it has four hydrophobic side chains and two phosphate groups. CL is present in energy-transducing membranes (for example, bacterial cytoplasmic membranes, mitochondrial inner membranes, and hydrogenosome membranes) in bacteria, plants, and animals and also as glycerol-di-archaetidyl-cardiolipin in archaea, most notably in halophiles (5, 6). In halophilic archaea, glycosyl-mono-archaetidyl-CL has been detected, but its function and synthesis are not known (7). Cardiolipin synthases (Cls) have been identified in bacteria and eukaryotes, and they belong to the phospholipase D (PLD) superfamily or to the CDP-alcohol phosphotransferase superfamily. Most Cls enzymes belonging to the PLD superfamily catalyze a reversible phosphatidyl group transfer from one phosphatidylglycerol (PG) molecule to another PG to form CL and glycerol. In contrast, Cls belonging to the CDP-alcohol phosphatidyltransferase superfamily use cytidine diphosphate-diacylglycerol (CDP-DAG) as the donor of the phosphatidyl group, which is transferred to a molecule of PG to form CL (1). In contrast to Eukaryotes and bacteria, the enzymes responsible for CL synthesis have not been previously identified in archaea (2).

Recently, Exterkate *et al.* (6) identified and characterized a cardiolipin synthase from the methanogen archaeon *Methanospirillum hungatei* (*Mh*Cls). The authors set out to identify candidate genes encoding putative Cls enzymes in archaea homologous to bacterial sequences. Homologs belonging to the PLD superfamily of Cls were found in halophilic and methanogenic archaea belonging to the phylum Euryarchaeota. The putative Cls from *M. hungatei* (*Mh*Cls) was selected for further study, because it was the only candidate present in this organism and because *M. hungatei* does not require extremophile growth conditions. The gene encoding *Mh*Cls was synthesized and recombinantly expressed in *E. coli*. In the absence of commercially available archaeal chiral substrate archaetidylglycerol (AG), both diastereomers were chemically synthesized and were found to be utilized by MhCls *in vitro*. Expression of *Mh*Cls in an *E. coli* Cls-null strain also led to CL formation. Based on this finding, further *in vitro* experiments using the bacterial substrates for CL synthesis were performed.

One of the surprising findings of their study was that *Mh*Cls is not only able to use two molecules of AG to form glycerol-di-archaetidyl-cardiolipin (as expected), but also was able to use two molecules of PG to form CL, just like bacterial Cls. In the presence of both AG and PG, bacterial–archaeal hybrid species could even be formed. When testing *Mh*Cls activity in the presence of PG and AG, surprisingly in addition to CL and glycerol-di-archaetidyl-CL, similar amounts of a hybrid glycerol-archaetidyl-phosphatidyl-CL were formed, indicating that there is no clear preference between the two lipid substrates and that substrate recognition only involves the polar headgroup of AG and PG. Indeed, in the reverse reaction using CL as a substrate, either water or glycerol could serve as the second substrate within the cell, leading to synthesis of both AG and PG, respectively. Finally, the authors used a number of other substrates to demonstrate the promiscuity of *Mh*Cls for various headgroups. *In vitro*, *Mh*Cls could utilize other substrates in the reverse reaction, including primary alcohols, a two-carbon-diol, the six-carbon polyol mannitol, or 1,3- propanediol with bulky methyl groups in the 2 position, leading to the formation of PG and respective phosphatidylalcohols. Even phosphatidylcholine and phosphatidylserine could be synthesized by the *Mh*Cls reverse reaction in the presence of choline and serine. Finally, although inefficiently, glycosyl-mono-archaetidyl-CL could be formed by *Mh*Cls from PG and monogalactosyl diacylglycerol, indicating that *Mh*Cls could form this naturally occurring lipid in archaea.

The study by Exterkate *et al.* opens new and exciting directions for lipid research. Having identified the gene encoding a glycerol-di-archaetidyl-CL synthase in archaea should allow for the possibility to create mutants deficient in this gene to study the function of this lipid (and possibly other CL-related lipids) in archaea under different growth conditions. However, these types of studies are not easy to perform, because many archaea are extremophiles, and therefore not easy to culture. Additionally, molecular biology techniques will have to be developed to construct these mutants.

Studies structurally comparing *Mh*Cls with other Cls of the PLD family could reveal why *Mh*Cls accepts bacterial and archaeal substrates. There is even a question as to whether well-studied Cls enzymes such as ClsA from *E. coli* would accept AG as a substrate. It is fascinating that one enzyme can synthesize both bacterial and archaeal lipids, indicating that the lipid divide might be less strict than previously thought. In this same direction, it was recently discovered that some bacteria encode a putative archaeal pathway for ether-bound isoprenoid membrane lipids in addition to the bacterial fatty acid membrane pathway. Recombinant expression of these enzymes in *E. coli* resulted in the formation of a “mixed archaeal/bacterial membrane” (8). In the presence of the appropriate substrates, *Mh*Cls can synthesize a surprising and impressive diversity of amphiphilic lipids. This promiscuity of *Mh*Cls could be further explored for bioengineering processes and could have applications in synthetic biology. New amphiphilic lipids with interesting and useful properties could be synthesized and characterized for various applications, such as the construction of synthetic membranes with engineered properties, taking advantage of the possibility to incorporate isoprene or fatty acid hydrophobic chains, and a plethora of functional headgroups.

## Conflict of interest

The author declares that he does not have a conflict of interest with the contents of this article.
